# Genome sequence surveys of *Brachiola algerae* and *Edhazardia aedis* reveal microsporidia with low gene densities

**DOI:** 10.1186/1471-2164-9-200

**Published:** 2008-04-29

**Authors:** Bryony AP Williams, Renny CH Lee, James J Becnel, Louis M Weiss, Naomi M Fast, Patrick J Keeling

**Affiliations:** 1 Canadian Institute for Advanced Research, Department of Botany, University of British Columbia, 3529-6270 University Boulevard, Vancouver, BC, V6T 1Z4, Canada; 2 Center for Medical, Agricultural and Veterinary Entomology, USDA/ARS, Gainesville, FL 32608, USA; 3 Department of Pathology, Division of Tropical Medicine and Parasitology, Albert Einstein College of Medicine, Bronx, New York 10461, USA

## Abstract

**Background:**

Microsporidia are well known models of extreme nuclear genome reduction and compaction. The smallest microsporidian genomes have received the most attention, but genomes of different species range in size from 2.3 Mb to 19.5 Mb and the nature of the larger genomes remains unknown.

**Results:**

Here we have undertaken genome sequence surveys of two diverse microsporidia, *Brachiola algerae *and *Edhazardia aedis*. In both species we find very large intergenic regions, many transposable elements, and a low gene-density, all in contrast to the small, model microsporidian genomes. We also find no recognizable genes that are not also found in other surveyed or sequenced microsporidian genomes.

**Conclusion:**

Our results demonstrate that microsporidian genome architecture varies greatly between microsporidia. Much of the genome size difference could be accounted for by non-coding material, such as intergenic spaces and retrotransposons, and this suggests that the forces dictating genome size may vary across the phylum.

## Background

Microsporidia are obligate intracellular eukaryotic parasites that have been found to infect members of all major animal lineages [1]. The many apparently "primitive" features of microsporidian cells led evolutionary biologists to suggest that they were an early-branching lineage of the eukaryotes [2,3], but molecular phylogeny has since shown that they are instead a derived relative of fungi [4,5]. In light of this, their seemingly primitive features have been re-evaluated as products of reduction and adaptation to life inside another cell [4-8].

One such feature that has attracted considerable attention is their highly reduced genomes. The genome size has been determined for numerous microsporidian species, and they range from 19.5 Mbp in *Glugea atherinae *to just 2.3 Mbp in *Encephalitozoon intestinalis*, the smallest eukaryotic genome known [9]. The two best-studied examples are the vertebrate parasite *Encephalitozoon cuniculi *(2.9 Mbp), the genome of which has been completely sequenced and encodes 1,997 protein-coding genes [10], and the insect parasite *Antonospora locustae *(5.4 Mbp) for which two sequence surveys are available [11,12]. These two species revealed just how microsporidian genomes had become so small compared with those of other eukaryotes. There has been a severe reduction in the number of genes in the genome, most likely a reflection of the fact that microsporidia are dependent on their hosts for many metabolic processes and import many compounds from their host. Furthermore the genes that remain are packed together very densely: intergenic spaces are minimal. In *E. cuniculi *there are no selfish elements and just 15 small introns. Genes in *E. cuniculi *are also shorter than their homologues in *Saccharomyces cerevisiae*, which is hypothesized to result from the small number of proteins within the cell, and a correspondingly smaller interaction network [10,13]. This extreme compaction appears to have resulted in a high level of gene order conservation between different species of microsporidia [12,14-16] and an unusually high level of overlapping transcription between adjacent genes [17,18].

Because the smallest microsporidian genomes are so unusual, they have garnered the greatest attention, and to date no large-scale survey of any larger genomes is available. This is unfortunate, because the form and content of these larger microsporidian genomes could differ from the smaller ones in many potentially interesting ways. On one hand they may contain a great many more genes, and could therefore reflect a greater cellular or metabolic complexity than the microsporidian parasites we presently know best. On the other hand these genomes may encode a great deal more non-coding DNA, which would have interesting implications for genome evolution within the group, and for why the smaller genomes are so compact. In other eukaryotes, it appears that variation in genome size on a relatively short evolutionary time scale is due to increased or decreased proportions of transposable elements in a genome [19]. There are hints that this may also be at least partially true in some microsporidian genomes where selfish elements have been found [20,21]. Most interestingly, a number of TY3/Gypsy retrotransposons have recently been described from the 15.3 Mbp genome of *Nosema bombycis *[22]. These elements are apparently flanked by areas of compacted genes that share a high level of synteny with *E. cuniculi*, perhaps suggesting an invasion of transposable elements into a compacted genome. More recently, the previously unsequenced subtelomeric areas of *E. cuniculi *have been investigated and found to contain a large family of proteins with at least 30 distinct members. This family of duplicated proteins is also found within other human infecting microsporidia [23], demonstrating that the expansion of gene families might be more common than previously thought.

Here, we describe low-redundancy genome sequence surveys of two distantly related microsporidia, *Brachiola algerae *(recently proposed to be renamed *Anncaliia algerae *[24]) and *Edhazardia aedis*. Both of these species have mosquitoes as their type hosts with the former having a very broad host range, including man, and the latter restricted to mosquitoes [25]. The genome size of *E. aedis *is not known, whereas the genome size of *B. algerae *has been estimated to be 15–20 Mbp [23]. In both species, we show that gene density is very low compared to that of the better-studied species of microsporidia. Specifically, we have found a considerable proportion of repetitive elements in both genomes, large stretches of non-coding DNA, and some evidence that the gene density may vary over the genome. These surveys open the possibility that microsporidian genomes are not universally compacted, large genomes do not necessarily encode significantly more genes than do the smaller genomes, and that dense genomes may sometimes revert to a gene-sparse state.

## Results and discussion

### General features of the sequence data

Two short-insert genomic libraries were constructed from *B. algerae *and a total of 219 clones fully or partially sequenced to yield 203,748 bp of non-overlapping sequence. A single *E. aedis *library was constructed and 290 sequence reads from 182 clones yielded 233,509 bp of non-overlapping sequence. Comparing these with the dense, gene-rich genome of *E. cuniculi *(Table 1) reveals a sharp contrast in the overall nature of the genomes. From the *B. algerae *survey, 34 genes with identifiable homologues in other organisms were identified, and a further 33 potential ORFs greater than 100 codons but with no recognizable similarity to any other gene were found, resulting in a protein-coding content of 11% identifiable coding sequence and 20% including putative ORFs. In *E. aedis*, only 25 identifiable protein-coding genes and 21 ORFs were found, pushing the range of coding sequence still lower, to 8% identifiable coding sequence or 14% if putative ORFs are included (Table 1). In contrast, 52% of the *E. cuniculi *genome consists of protein-coding sequences with recognizable similarity to other genes, and the proportion of coding sequence is 87% when ORFs are included [10]. The gene density of *A. locustae *is similar to that of *E. cuniculi *[12], as are small regions of other microsporidian genomes that have been sampled [20]. Overall, the gene-densities of *B. algerae *and *E. aedis *are about an order of magnitude lower than other microsporidia that have been examined to date.

**Table 1:** Summary of the data compared to the genome of *E. cuniculi*

	***E. cuniculi *[10]**		***B. algerae***		***E. aedis***	
	**bp**	**%**	**bp**	**%**	**bp**	**%**

**Total sequenced**	2,497,519		203,748		233,509	
**coding**	2,180,498	87	41153(67)	20	33,617 (46)	14
**hits**	1,320,216	61	22260 (34)	54	19,099 (25)	57
**ORFS**	860,274	39	18893 (33)	46	14,518 (21)	43
**Non-coding**	317,029	13	162595	80	199,892	86

Numbers in brackets indicates the number of genes or gene fragments in each sample. The percentages refer to the proportion of each sequenced class relative to the total amount sequenced.

The overall GC content for both *B. algerae *(24%) and *E. aedis *(25%) is also significantly lower than that of *E. cuniculi *(47%). Not surprisingly, the GC content in the coding regions is slightly higher: 28% for *B. algerae *and 31% for *E. aedis*. A smaller sequence survey from *Spraguea lophii *with a genome size of 6.2 Mb has revealed a bias of 28% [20,26]. There is therefore no obvious correlation between genome size and drift towards low GC content in microsporidia, and similarly no pronounced lineage-specific bias.

### Presence of transposable elements

In contrast to the *E. cuniculi *genome, which does not encode any selfish genetic elements, fragments of reverse transcriptase or complete retrotransposons have been reported in the genomes of *N. bombycis*, *V. corneae *and *S. lophii *[20-22], and repeated sequences suggested to be mobile were reported in *N. bombycis *and *N. costelytrae *[27]. The *V. corneae *reverse transcriptase is closely related to a human LINE sequence, and both the *N. bombycis *and *S. lophii *retrotransposons have sequence similarity to each other and to Ty3/gypsy retrotransposons.

In both *B. algerae *and *E. aedis *surveys we found extensive evidence of numerous transposable elements (Table 2). The *E. aedis *fragments all share high similarity to Ty3/gypsy retrotransposons from the *N. bombycis *and *S. lophii*. Nine of the seventeen fragments of putatively selfish elements identified in *B. algerae *are also members of the same family, and once more also share a high degree of similarity to the *N*. *bombycis *and *S. lophii *elements. The remaining fragments from *B. algerae *were similar to hypothetical proteins resembling transposases.

**Table 2:** List of hits to suspected transposable elements:

**Top Blast hit annotation**	**Accession no.**
***Brachiola algerae***	

Pol polyprotein *Nosema bombycis*	91176517
Pol polyprotein *Nosema bombycis*	91176521
Pol polyprotein *Nosema bombycis*	91176521
Pol polyprotein *Nosema bombycis*	91176521
Pol polyprotein *Nosema bombycis*	91176521
Pol polyprotein *Nosema bombycis*	91176523
Pol polyprotein *Nosema bombycis*	91176523
Pol polyprotein *Nosema bombycis*	91176525
Pol polyprotein *Nosema bombycis*	91176525
Transposase, putative *Acaryochloris marina*	158337326
Predicted protein, *Nematostella vectensis*	156394155
Conserved hypothetical protein *Akkermansia muciniphila*	166832600
*Neisseria meningitidis *IS1016 transposase	161869234
*Caenorhabditis briggsae *hypothetical protein CBG18017	157775203
*Caenorhabditis briggsae *hypothetical protein CBG18017	157775203
*Caenorhabditis briggsae *Hypothetical protein CBG00277	157771110
*Caenorhabditis briggsae *Hypothetical protein CBG21915	157749299

***Edhazardia aedis***	

Pol polyprotein *Nosema bombycis*	91176525
6 different Pol polyproteins *Nosema bombycis*	91176519
Pol polyprotein *Nosema bombycis*	91176525

The level of similarity between the Ty3/gypsy elements from *N. bombycis, S. lophii, E. aedis *and *B. algerae *and the fact that the host groups for these four species are not closely related, is strongly suggestive that an ancestral family of retrotransposons existed in the common ancestor of these microsporidia. In molecular phylogenies of microsporidia, the true *Nosema*-group is consistently found to be a sister-lineage of the *Encephalitozoon*-group to the exclusion of lineages that include *E. aedis*, *B. algerae *and *S. lophii *[28-30]. If the Ty3/gypsy retroelements identified here are ancestral to the genomes where they have been found, it means that it was also ancestral to *E. cuniculi *and must have been completely purged from its genome. This raises some curious questions about the *N. bombycis *genome. Here, the Ty3/gypsy retrotransposons were found to be nested within blocks of compacted genes that were often conserved in order with homologues from other microsporidian genomes [22]. Based on this it was suggested that the elements could have invaded a compact genome, and perhaps later facilitated some genomic rearrangements [22]. Reconciling the ancient presence of these elements with the nature of the *N. bombycis *genome is complicated. It is possible that the ancestral genome contained many such elements and had a low gene density. This genome could have subsequently compacted in several lineages, some of which lost the retroelements as part of the compaction process (e.g., *E. cuniculi*), while others kept them and compacted the genome around them (e.g., *N. bombycis*). It is also possible that compaction happened in an earlier common ancestor of some of these lineages and that certain genomes have 're-expanded'. In either event, the retention of large numbers of selfish elements in an otherwise compact genome is of interest, as one might expect that compaction would be strongly inclined to lead to the loss of non-coding material such as selfish elements. It serves to illustrate the way compaction affects different aspects of the genome in different lineages, another possible example being the differential loss or retention of introns in relict nucleomorph genomes [31,32].

### Gene density, order, and size

The small number of genes identified and the large continuous stretches of non-coding sequence in both surveys lead to the obvious conclusion that the gene-density of these genomes is much lower than those of *E. cuniculi *or *A. locustae*. The average intergenic distances in these genomes cannot readily be determined since few have been completely sequenced. In *B. algerae *four clones encoded two genes and the distances between them are 108, 206, 276, and 552 bp. In *E. aedis *a single clone encoded two adjacent genes, and the intergenic spaces between them is 1,324 bp. At the same time, the largest continuous stretches of sequence from which we could identify no genes were 2,412 (or 2,943 in a likely subtelomeric region next to an SSU gene) and 2,068 bp in *B. algerae *and *E. aedis*, respectively. The average distance between genes in *E. cuniculi *and *A. locustae *samples is 129 and 211 bp [10,12]. From the existing data it seems likely that the average distance between genes in *B. algerae *and *E. aedis *is much larger than that of either of the well studied microsporidian genomes, and that the density across at least the *B. algerae *genome may be more heterogeneous.

**Figure 1:**
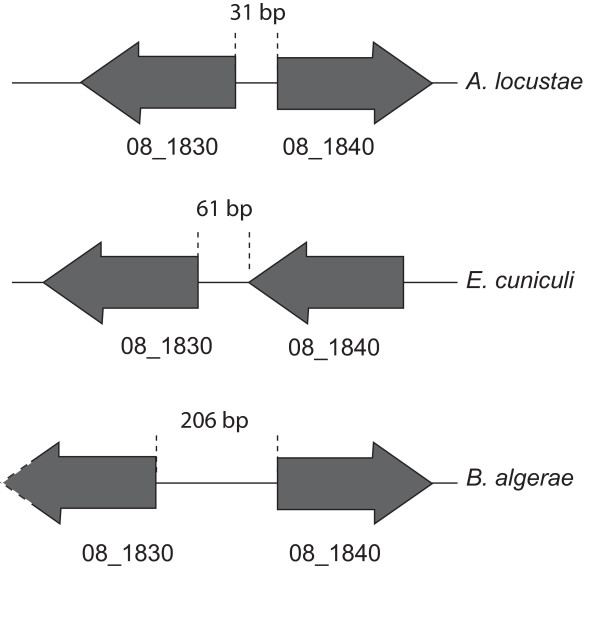
**Area of conserved gene synteny between three species of microsporidia**. A fragment of the *B. algerae *genome aligned to corresponding regions from *E. cunculi *and *A. locustae*. The gene order, but not orientation, is conserved. Arrowheads indicate gene orientation and dashed white line indicates incomplete gene sequences. Intergenic space lengths are indicated.

Of the pairs of adjacent genes we identified, one *B. algerae *pair is also adjacent in both *A. locustae *and *E. cuniculi *(Figure 1) (the pair separated by 206 bp in *B. algerae*). It has previously been shown that the order of gene pairs in *A. locustae *and *E. cuniculi *is highly conserved, and this has been hypothesized to be related to the compaction of the genome [12]. The conservation of one of four *B. algerae *gene pairs suggests that areas of this genome may be under similar constraints. If the conservation of genome order is related to compaction, this also suggests that the compacted state may have existed in the ancestor of *B. algerae*, *E. cuniculi *and *A. locustae*, which is consistent with phylogenies that suggest some relationship between *B. algerae *and *A. locustae *[[25] and unpublished data].

**Figure 2:**
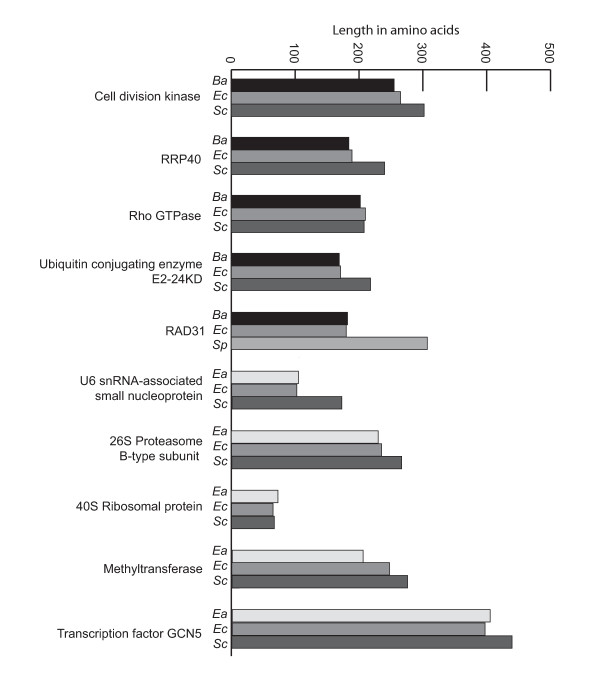
**Comparison of microsporidian and yeast protein lengths**. The number of codons for all full-length proteins found within *B. algerae *(*Ba*) and *E. aedis *(*Ea*) sequence surveys compared to homologues from *S. cerevisiae *(*Sc*) (or *Schizosaccahromyces pombe *(*Sp*) in cases where *S. cerevisiae *does not encode a homologue).

In addition to being densely packed, *E. cuniculi *genes have also been shown to be shorter on average than homologues in the *S. cerevisiae *genome [10]. This has been discussed in the context of genome compaction, but also suggested to be the result of a reduction in the number of proteins in the cell, which leads to smaller interaction networks, which in turn allows proteins to reduce their number or complexity of interacting domains [13]. In yeast, it has been shown that there is a correlation between protein size and connectivity, with larger proteins displaying a greater number of interactions [33]. We examined the only five full-length genes identified in the *B. algerae *survey with homologues in yeast and found that all five were shorter than *S. cerevisiae *homologues, and more surprisingly most were also shorter than the *E. cuniculi *homologues (Figure 2). Similarly, from the *E. aedis *survey, only five full-length genes were found (Figure 2), and four of these were shorter than *S. cerevisiae *homologues, and comparable in size with the *E. cuniculi *homologues. The sole *E. aedis *protein predicted to be larger than the yeast counterpart encodes a ribosomal protein (Figure 2). Given that these genomes are not compacted, it suggests that proteins are either shorter due to a reduced proteome complexity, or that they had an ancestor with a compacted genome, or both.

### Gene content and coding capacity

There is no experimental estimate of the *E. aedis *genome size, but the *B. algerae *genome has been estimated to be between 15–20 Mbp by pulsed-field gel electrophoresis [23]. This is much larger than the genomes of either *E. cuniculi *(2.9 Mbp) or *A. locustae *(5.4 Mbp). As we show, much of the genome size difference can be attributed to the significantly lower gene-densities of *B. algerae *and *E. aedis*. However, it is still possible that one or both of these genomes is also larger because it contains more genes than *E. cuniculi*.

The *E. cuniculi *genome clearly under went a massive gene loss relative to other eukaryotes, but this gene loss may have been ancestral to microsporidia. If this were the case we would expect to find few genes in *E. aedis *and *B. algerae *that are present and conserved in other eukaryotes that are not also present in *E. cuniculi*. That is to say that the *B. algerae *and *E. aedis *genomes would not have more conserved genes than the pool remaining in the ancestral microsporidian after this gene loss event.

Our sampling of *E. aedis *and *B. algerae *genomes shows that this scenario is quite possible. Of the protein-coding genes with identifiable homologues in some other genome that we found in *B. algerae *(34 cases) and *E. aedis *(25 cases), every one is also present in *E. cuniculi *(Table 3). Given the sample size, it is likely that either genome could contain some genes found in other organisms but not *E. cuniculi*, but it is unlikely that they are abundant. This lack of excess conserved gene homologues is of interest because it implies that the large-scale gene loss characteristic of *E. cuniculi *took place relatively early in microsporidian evolution, in the ancestor of *E. cuniculi*, *A. locustae*, *E. aedis *and *B. algerae*.

**Table 3:** List of genes identified by BLAST search

***Brachiola algerae***		***Edhazardia aedis***	
**Hit**	***E. cuniculi *locus**	**Hit**	***E. cuniculi *locus**

Cell Division Kinase	08_0230	14-3-3 Protein 1	03_1010
Coatomer coat delta	08_0340	26S proteasome beta-type subunit	05_0290
DNA directed RNA pol	01_0600	40S ribosomal protein S28	09_1275
DNA ligase	02_1220	60S ribosomal protein L8	01_0310
DNA mismatch repair	11_1260	Aldose Reductase	01_0970
Dnm1	01_1210	DNA Mismatch Repair Protein	05_0300
*E. cuniculi *hypothetical protein	01_0390	Endochitinase	09_1320
*E. cuniculi *hypothetical protein	04_0270	*E. cuniculi *hypothetical protein	03_0870
*E. cuniculi *hypothetical protein	05_1460	*E. cuniculi *hypothetical protein	05_1000
*E. cuniculi *hypothetical protein	06_0970	*E. cuniculi *hypothetical protein	05_1080
*E. cuniculi *hypothetical protein	07_0810	*E. cuniculi *hypothetical protein	09_1690
*E. cuniculi *hypothetical protein	08_1830	Myosin heavy chain	09_1970
*E. cuniculi *hypothetical protein	08_1840	NIFS-like protein	11_1770
*E. cuniculi *hypothetical protein	09_0300	Phospholipid-transporting ATPase	09_1440
*E. cuniculi *hypothetical protein	09_1240	Putative methyltransferase	05_0950
*E. cuniculi *hypothetical protein	10_1360	RING-finger-containing ubiquitin ligase	07_0330
*E. cuniculi *hypothetical protein	11_0260	Similarity to oxidoreductase	11_1070
GPI Anchor Biosynthesis	09_1210	SSU gene	
Hsp70	02_0100	Topoisomerase 1	06_1520
Isopentyl pyrophosphate δ isomerase	02_0230	TPR domain hypothetical protein	09_1180
LSU gene		Transcriptional activator	10_1430
Pelota protein	03_1380	Translation initiation factor IF-2P	09_0070
Phenylalanine tRNA synthase	07_1660	Trehalose-6-phosphate synthase	01_0870
RAD31 DNA damage tolerance	08_0460	U6 snRNA-associated small RNP	05_1310
RAS-like GTP binding protein	10_0350	UTP glucose-1-phosphate uridyltransferase	03_0280
Septin	09_0820	Vacuolar protein sorting-associated protein	03_0900
SER/THR protein kinase	08_1620		
Signal Recognition Particle	04_0980		
SSU gene			
Syntaxin	05_0820		
TFIID 111 KDa	01_0760		
TFIID 72/90 KDa	11_1750		
TFIID 150 kDa	09_0090		
TFIID I	04_1440		
U5 Associated snRNP	11_0870		
Ubiquitin Conjugating Enzyme E2	08_0860		

However, the *B. algerae *survey consisted of 20% coding sequence, so taking into account the range or estimated genome sizes for *B. algerae *(15–20 Mbp), this suggests between 2,786 and 3,714 genes in the *Brachiola *genome (assuming an average gene length of 1,077 as in *E. cuniculi*). The discrepancy between this predicted coding capacity of *B. algerae *and the observation that all the recognizable genes we sampled are shared with *E. cuniculi *could be explained in many ways. First, our sample may be biased to gene-encoding regions, and this would lead to an overestimate of the gene-density. Second, a large number of lineage-specific ORFs could skew the estimate, but the proportion of ORFs we found (47% and 43% for *B. algerae *and *E. aedis*, respectively) is similar to that found in *E. cuniculi *(39%). This issue is also complicated by the fact that we identified several *E. cuniculi *"ORFs" in our sample, and therefore the proportions of putative ORFs is changing. Third, the genome size estimates may be wrong. Lastly, it is possible that there are many more than 2,000 genes in these organisms, but that the excess is mostly due to recent duplications. We did not sample any duplicates in either genome, though we did find areas of repeats amongst the non-coding areas in both *B. algerae *and *E. aedis*. If gene duplications are common, the genome could contain more genes without an increased complexity in the proteome.

### Evolution of genome compaction in microsporidia

**Figure 3:**
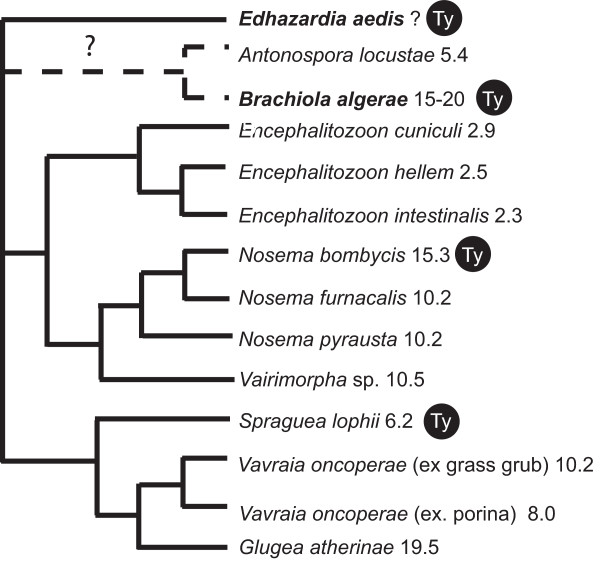
**Schematic consensus of microsporidian phylogenetic relationships**. Microsporidian relationships from a consensus of published SSU phylogenies [28, 30, 40] and concatenated tubulin genes (unpublished data). Genome sizes are labeled and the reported presence of Gypsy/Ty transposons is indicated by (Ty).

Though the phylogenetic relationships of major microsporidian lineages are not well resolved, phylogenies of rRNA [28] and concatenated tubulin genes (unpublished data) suggest a relationship between *B. algerae *and *A. locustae *to the exclusion of *E. aedis *or *E. cuniculi *(Figure 3). This raises interesting questions about whether microsporidian genomes have compacted more than once during the diversification of the phylum, or if some have re-expanded from a compacted state.

An obvious factor in the dynamics of genome size is transposable elements. One could imagine a genome expanding due to the invasion of such elements, and indeed many such elements have been found in *N. bombycis *[22] and now *B. algerae *and *E. aedis*. However, the majority of these elements are closely related members of the Ty3/gypsy family. Therefore genome expansion cannot be entirely due to an invasion since the elements must have existed in the common ancestor and been purged from *E. cuniculi *and possibly other compacted microsporidian genomes. It also remains to be seen if these genomes are substantially heterogeneous. It is possible that many genes do exist in relatively compact regions while other regions are dominated by non-coding sequence. An extreme version of such a situation is seen in the small and compacted genome of the picoplankton *Ostreococcus tauri*. Here most chromosomes in the genome show a high gene density but 2 chromosomes out of 20 contain 77% of the transposons identified in the genome [34].

A second factor that has been hypothesized to affect genome size is cell size. A correlation between genome size and cell size has been observed in eukaryotes generally [35] and microsporidia specifically [36]. However in the microsporidia, variation in cell size in the different life stages can confound correlations between genome size and cell size. As a rough correlation though, the genome size of *B. algerae *is estimated at 15–20 Mb and that of *A. locustae *is reported to be 5.4 Mb [37], whereas the spores of both *B. algerae *and *A. locustae *are of comparable sizes [38,39] suggesting, in this case, that cell size is not necessarily a factor. A further consideration is whether the complexity of the life cycles of different microsporidia is reflected in genome size and gene number. Both *A. locustae *and *E. cuniculi *have a simple life cycle with monomorphic spores and are restricted to a narrow host range. *Edhazardia aedis *has a more complex life cycle with multiple spore types and must be adapted to both the larval and adult stage of the mosquito. *Brachiola algerae*, known to have a larger genome, has a simple life cycle with monomorphic spores, but has a broad host range and can infect both mammals and insects.

## Conclusion

The *E. cuniculi *genome is a model for compacted nuclear genomes, but potentially not a good model for microsporidian genomes generally. We have shown that the genomes of *B. algerae *and *E. aedis *are structured very differently: they have large proportions of non-coding sequence and many transposable elements, resulting in a very low and perhaps variable gene-density compared with *E. cuniculi*. The sample of identifiable genes found in the surveys, and the proportions of these genes shared with *E. cuniculi *both suggest that the complexity of the proteome is not the major factor contributing to genome size variation. The phylogeny of microsporidia suggests multiple events of compaction and/or expansion, which raises interesting questions about what forces the genomes to compact so severely, and why such a force would then cease to operate on the genome.

## Methods

### Microsporidia, genomic DNA extraction, and genomic library construction

6.1 × 10^7 ^uninucleate *Edhazardia aedis *spores harvested from *Aedes aegypti *larvae were ruptured by glass bead-beating, and spores were examined for breakage via light microscopy. *E. aedis *genomic DNA was purified by the standard phenol-chloroform method and served as template for whole genome rolling-circle amplification using Genomiphi (Amersham). 4.5 μg of amplified *E. aedis *genomic DNA was sheared, blunt end-repaired, and cloned into pCR4Blunt-TOPO (Invitrogen) according to the manufacturer's specifications. 182 different *E. aedis *clones with an average length of 1,283 bp were end-sequenced using ABI Big Dye 3.1 chemistry. Six different *E. aedis *library clones containing coding, non-coding, or transposable segments were checked for chimeric sequence by PCR of non-Genomiphi-treated *E. aedis *genomic DNA. From this, successful amplification of fragments between 250 and 450 bp did not support the idea of chimeras being present in the Genomiphi-created *E. aedis *genomic library.

DNA was extracted from 2 × 10^7^germinated *Brachiola algerae *spores (a strain originally isolated the mosquito *Anopheles stephensi*). Spores were cultivated *in vitro *in RK13 rabbit kidney cells at 30C in 5% CO_2_, purified and germinated by incubation in 0.3% H_2_O_2 _for 16 hours at 25°C. The germinated spores were concentrated by centrifugation suspended in 100 μg/ml Proteinase K-PBS and incubated for 15 min at 65°C. DNA was extracted using phenol-chloroform, followed by ethanol precipitation. DNA was then dissolved in TE buffer and stored at -70°C until use. A sample of purified *Brachiola algerae *DNA was amplified using Genomphi (Amersham) to produce 10 μg of DNA and another sample of 1.7 μg of purified DNA was processed directly to make two separate libraries. DNA was sheared, blunt-ended and cloned as described for *E. aedis *above. A respective 64,433 and 140,051 bases from the Genomiphi and non-Genomiphi treated libraries were sequenced with ABI Big Dye 3.1. This gave a total of 203,748 non-overlapping bases of sequence in 181 contigs with an average length of 1,125 bp.

Areas of six representative *B. algerae *clones from the Genomiphi-amplified DNA library were reamplified by PCR from genomic DNA to confirm that the Genomiphi process had not amplified chimeric sequences. Primers were designed to areas of 6 clones. These fragments were between 525 and 1000 base pairs and included non-coding areas, putative transposases, transposons, protein-coding genes, and an SSU gene area.

Contigs were analysed by BlastX and BlastN to sequences in GenBank. Open reading frames were considered significantly similar if E values were less than 0.00001. Contigs were further searched for stretches of nucleotides coding for sequences of at least 100 amino acids, and these were considered ORFs.

New sequences were deposited in GenBank under accession numbers ET437577–ET437812 and ET437979–ET437981 (*E. aedis*) and ET223031–ET223211 (*B. algerae*).

## Authors' contributions

BAPW and RCHL constructed libraries, sequenced clones, analysed data and drafted the paper, JJB and LMW contributed microsporidian material and to the writing of the paper. NMF and PJK conceived of the study, analysed data and drafted the paper.
